# Morphological Profiling Identifies a Common Mode of Action for Small Molecules with Different Targets

**DOI:** 10.1002/cbic.202000381

**Published:** 2020-07-24

**Authors:** Tabea Schneidewind, Alexandra Brause, Axel Pahl, Annina Burhop, Tom Mejuch, Sonja Sievers, Herbert Waldmann, Slava Ziegler

**Affiliations:** ^1^ Max-Planck Institute of Molecular Physiology Department of Chemical Biology Otto-Hahn-Strasse 11 Dortmund 44227 Germany; ^2^ Technical University Dortmund Faculty of Chemistry and Chemical Biology Otto-Hahn-Strasse 6 Dortmund 44227 Germany

**Keywords:** antiproliferative, cell cycle, iron chelators, mode of action, phenotypic profiling

## Abstract

Unbiased morphological profiling of bioactivity, for example, in the cell painting assay (CPA), enables the identification of a small molecule's mode of action based on its similarity to the bioactivity of reference compounds, irrespective of the biological target or chemical similarity. This is particularly important for small molecules with nonprotein targets as these are rather difficult to identify with widely employed target‐identification methods. We employed morphological profiling using the CPA to identify compounds that are biosimilar to the iron chelator deferoxamine. Structurally different compounds with different annotated cellular targets provoked a shared physiological response, thereby defining a cluster based on their morphological fingerprints. This cluster is based on a shared mode of action and not on a shared target, that is, cell‐cycle modulation in the S or G2 phase. Hierarchical clustering of morphological fingerprints revealed subclusters that are based on the mechanism of action and could be used to predict target‐related bioactivity.

## Graphical abstract

**Route planner**: We explored a morphology‐based cluster around the iron chelator deferoxamine by using the cell painting assay and identified annotated compounds with different targets but a similar mode of action. This cluster can be used to predict modes of action for unexplored small molecules, thus highlighting the value of morphological profiling to explore the bioactivity of compound collections.

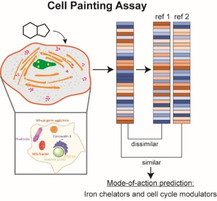

## Introduction

Phenotypic screening for perturbed or restored cellular phenotypes is a powerful approach to identifying small‐molecule bioactivity. However, it is limited to the phenotype of interest in a given assay, and wider coverage of bioactivity space requires the exposure of small molecules to multiple phenotypic assays. This limitation can be overcome by morphological profiling, for example, in the cell painting assay (CPA).^[1]^ Cell painting uses six dyes for selective staining of different cell organelles or components, followed by high‐content imaging and analysis to extract morphological features and to generate a characteristic morphological fingerprint for a query compound. Comparison with fingerprints recorded for a set of reference compounds with known target(s) or mode of action (MoA) is employed as measure for possible biological similarity.^[2]^ The unbiased nature of morphological profiling allows the identification of different bioactivities in a single assay directly delivering a MoA or target hypothesis if similarity to a reference is given. Another limitation of commonly applied target identification methods,^[3]^ such as affinity‐based chemical proteomics, is the restriction to protein targets. In contrast, phenotypic profiling can suggest and identify a MoA based on biological similarity alone. This is particularly important for small molecules with nonprotein targets, or which may have both, protein and nonprotein targets not identified during compound development.[^2a^, ^4^] Targeting nonprotein biomolecules like lipids,^[5]^ DNA,^[6]^ or RNA^[7]^ evolved as a new research area in the last years. Profiling approaches like morphological profiling can also rapidly suggest a MoA for nonprotein targeting agents.[^2a^, ^8^] Moreover cheminformatic methods for target identification, often employed subsequent to phenotypic screening or morphological profiling, are usually centered on chemical and structural similarity, and structure and sequence similarity among potential target proteins[^4d^, ^9^] limiting the identification of a nonprotein target.

Here we describe that morphological profiling using the cell painting assay enables the identification of a common MoA for compounds with similar morphological fingerprints, but which have different annotated protein targets or which might not target proteins at all. CPA revealed high morphological fingerprint similarity (biosimilarity) across structurally very different nonprotein targeting iron chelators (exemplified by deferoxamine, DFO) and compounds that induce cell‐cycle arrest in the S or G2 phase. This biosimilarity is due to impairment of cell‐cycle progression as a common denominator of iron chelators and S/G2 phase regulators that do not chelate iron as previously reported.^[8]^ This shared MoA is based on the physiological response upon iron depletion. Various enzymes involved in DNA synthesis require iron as a cofactor^[10]^ and iron chelators are known to inhibit cell proliferation and induce cell‐cycle arrest in G1/S‐phase.^[11]^ Based on their morphological fingerprints, these compounds define a cluster. We employed this cluster to identify biosimilar small molecules in our library of annotated agents as well as within our in‐house collection of unexplored compounds. Our results expand the set of reference compounds that, based on a shared MoA, belong to this cluster and identify novel modulators of the cell‐cycle G1/S‐phase.

## Results and Discussion

Structurally different compounds with different annotated cellular targets can cause shared physiological responses. These compounds define a cluster based on their mode of action. Comparison of morphological fingerprints to reference compounds allows target/MoA prediction for uncharacterized compounds with unknown activity. We employed morphological profiling to identify compounds that do not target proteins as this activity is rather difficult to identify with commonly applied target/MoA identification methods. Therefore, we focused on the iron chelator deferoxamine and identification of a cluster of similar reference compounds with the desired MoA that would afterwards allow to identify novel compounds with shared MoA based on fingerprint similarity.

### Morphological profiling of deferoxamine (DFO)

**Figure 1:**
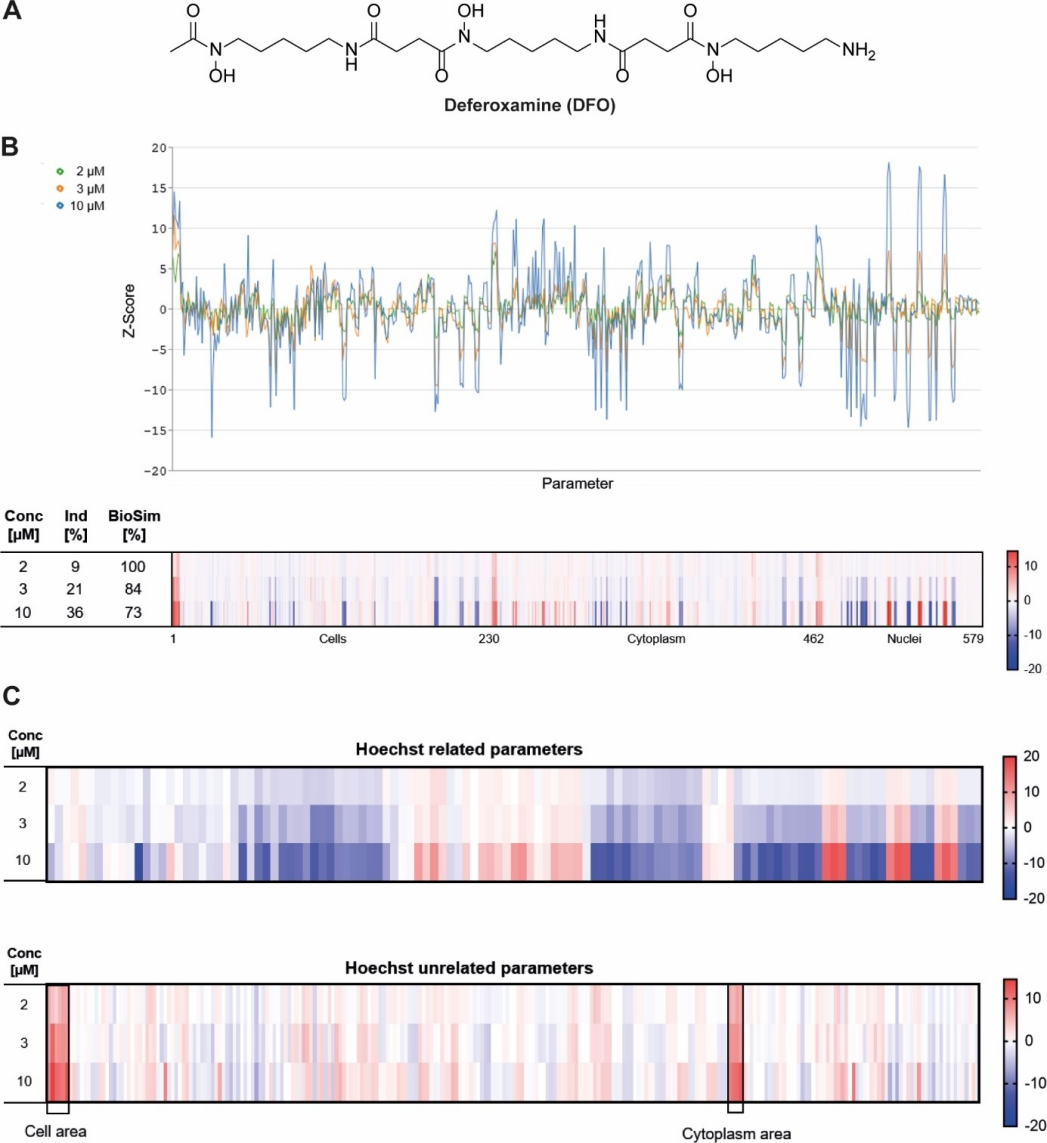
Morphological profiling of deferoxamine. A) Structure of DFO. B) Fingerprints determined for DFO at different concentrations visualized as line plots and a heatmap. The top line of the heatmap profile is set as a reference fingerprint (100 % biological similarity, BioSim) to which the following fingerprints are compared. The set of 579 parameters is divided in parameters related to the cell (1–229), cytoplasm (230–461) and nuclei (462–579). Values were normalized to the DMSO control. Blue: decreased parameter, red: increased parameter. C) Nucleus (Hoechst)‐related and unrelated parameters.

Deferoxamine (Figure 1A) is a hexadentate siderophore from *Streptomyces pilosus* that has high affinity to Fe^III^ and is clinically used in the treatment of iron‐overload diseases.[^10b^, ^12^] To explore the usefulness of CPA for prediction of iron‐chelating activity based on morphological fingerprint similarity (biosimilarity), we exposed human osteosarcoma U‐2OS cells to different concentrations of DFO and generated morphological fingerprints.^[13]^ U‐2OS cells are large, flat, grow in monolayer and adhere well to plastic and, thus, are especially well suitable for imaging.^[14]^

579 parameters were deduced from the high‐content analysis to generate the fingerprints and to calculate the *induction* (i. e., the number of significantly changed parameters [median absolute deviation of the parameter value >±3] in comparison to the vehicle control [%]) as a measure of bioactivity.^[2b]^ Fingerprints were compared by means of a *biosimilarity* score (*BioSim* [%], see the Supporting Information for further details) determined at different concentrations, which revealed a concentration‐dependent increase in induction, that is, from 9 % at 2 μM to 36 % at 10 μM, at high biosimilarity (BioSim ≥73 %; Figure 1B). Compared to the vehicle control, predominantly nucleus (Hoechst)‐related parameters were altered. In addition, the cell and the cytoplasm area increased dose dependently (Figure 1C).

### Identification of references with high biosimilarity to DFO

**Figure 2:**
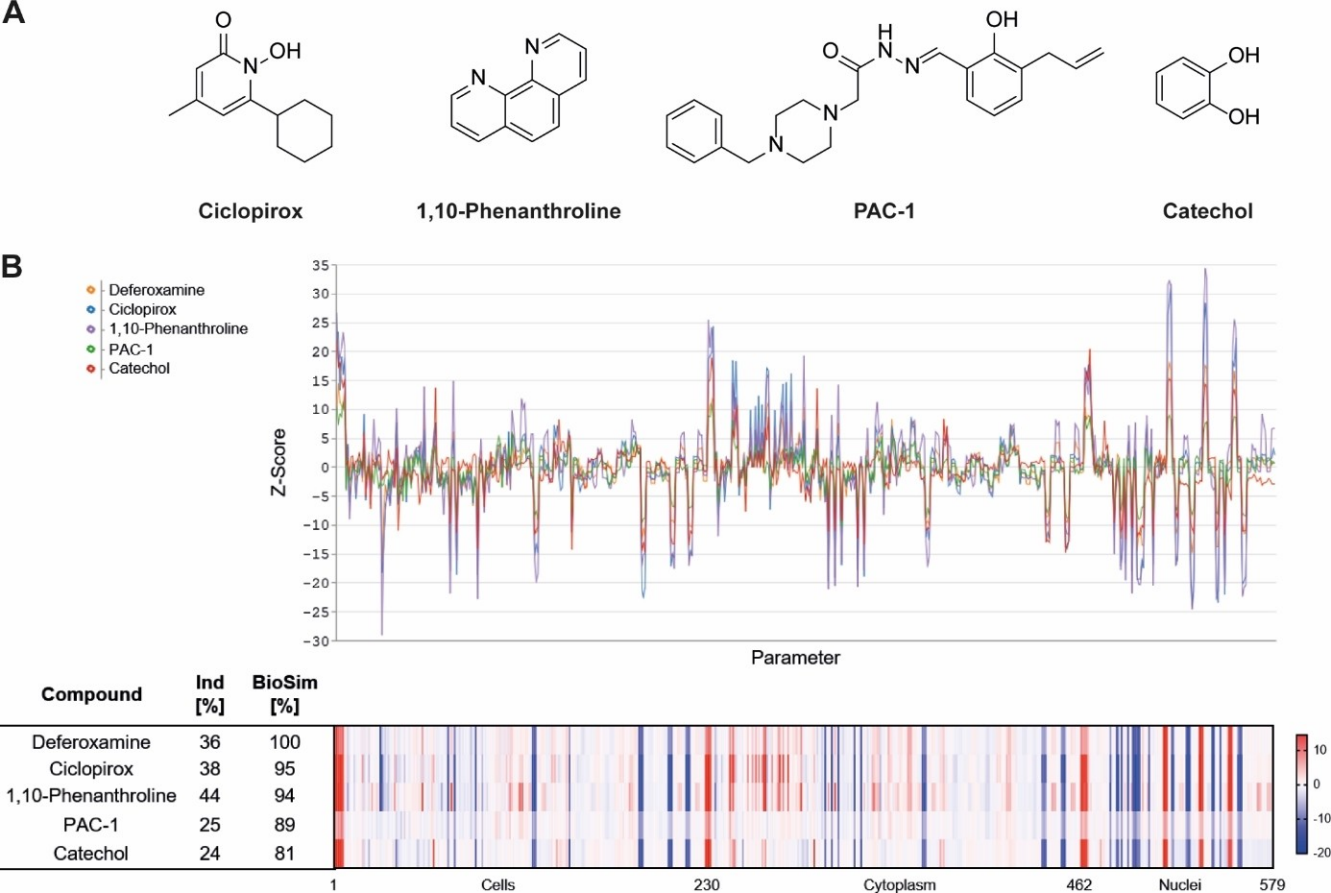
Morphological profiling of references linked to iron chelation that shared high biosimilarity to DFO. A) Structures of annotated iron chelators. B) Fingerprints of iron chelators at 10 μM visualized as line plots and heatmap. The top line of the heatmap profile is set as a reference fingerprint (100 % BioSim) to which the following fingerprints are compared. The set of 579 parameters is divided in parameters related to the cell (1–229), cytoplasm (230–461) and nuclei (462–579). Values were normalized to the DMSO control. Blue: decreased parameter, red: increased parameter.

3580 reference compounds were profiled in the cell painting assay with the goal to employ the resulting morphological fingerprints for target‐/MoA prediction for small molecules with unknown bioactivity. Amongst others, the reference set contains the Library of Pharmacologically Active Compounds (LOPAC), libraries of kinase inhibitors and the Prestwick Chemical Library. Using the morphological fingerprint of DFO as a query profile we identified several metal chelating agents among the reference compounds with the highest biosimilarity (>80 %). The metal ion chelators ciclopirox^[15]^ and 1,10‐phenanthroline^[16]^ (Figure 2A) shared high biosimilarity (95 and 94 %, respectively) to DFO (10 μM; Figure 2B). The fingerprint for the compound PAC‐1 (Figure 2A and B), which is annotated as a procaspase‐3 activator, was 89 % biosimilar to DFO. However, PAC‐1 activates procaspase‐3 by chelating zinc ions^[17]^ and iron‐chelating activity was recently reported.^[18]^ In addition, the fingerprint for the iron(III)‐selective ligand catechol (Figure 2A)[^10a^, ^10b^] showed 81 % similarity to the morphological fingerprint of DFO (Figure 2B). Most metal ion chelators can complex different metal ions and are, thus, rarely specific, in particular regarding Fe^2+^, Cu^2+^ and Zn^2+^.^[19]^ DFO has very high selectivity for Fe^III^ and chelates bivalent metal ions with substantially lower affinity.^[20]^ The metal‐chelating agents that are biosimilar to DFO have the highest affinity for ferrous and ferric ions (Table S1 in the Supporting Information). Therefore, the fingerprint similarity of the metal chelators to DFO is attributed to iron chelation and most likely not to complexation of other metal ions.

This finding demonstrates that iron chelators form a CPA cluster that, in principle, can be successfully employed for the detection of iron chelators and most likely for the identification of novel iron complexing agents.

Cheminformatic methods offer an orthogonal, *in silico* approach to predict bioactivity for small molecules, whereby the bioactivity information extraction and (re)assignment rest on known ligand‐target interactions and chemical and structural similarity of ligands and target proteins.[^4d^, ^4e^] These approaches are mostly centered on drug (small molecule)‐protein pairs. However, compounds can modulate biomolecule classes that are not proteins.[^4a^, ^4b^, ^4c^] Not unexpectedly, several web‐based cheminformatics tools did not predict the target of DFO, ciclopirox and 1,10‐phenanthroline (Tables S2 and S3). Only the PASS algorithm^[21]^ and SuperPred^[22]^ suggested an iron chelation activity (i. e., *iron antagonist*) for DFO. This calls for extension of the drug‐target space that is considered by cheminformatic approaches to nonprotein targets to facilitate MoA prediction early on in the target or MoA identification process for nonprotein targeting compounds.

**Figure 3:**
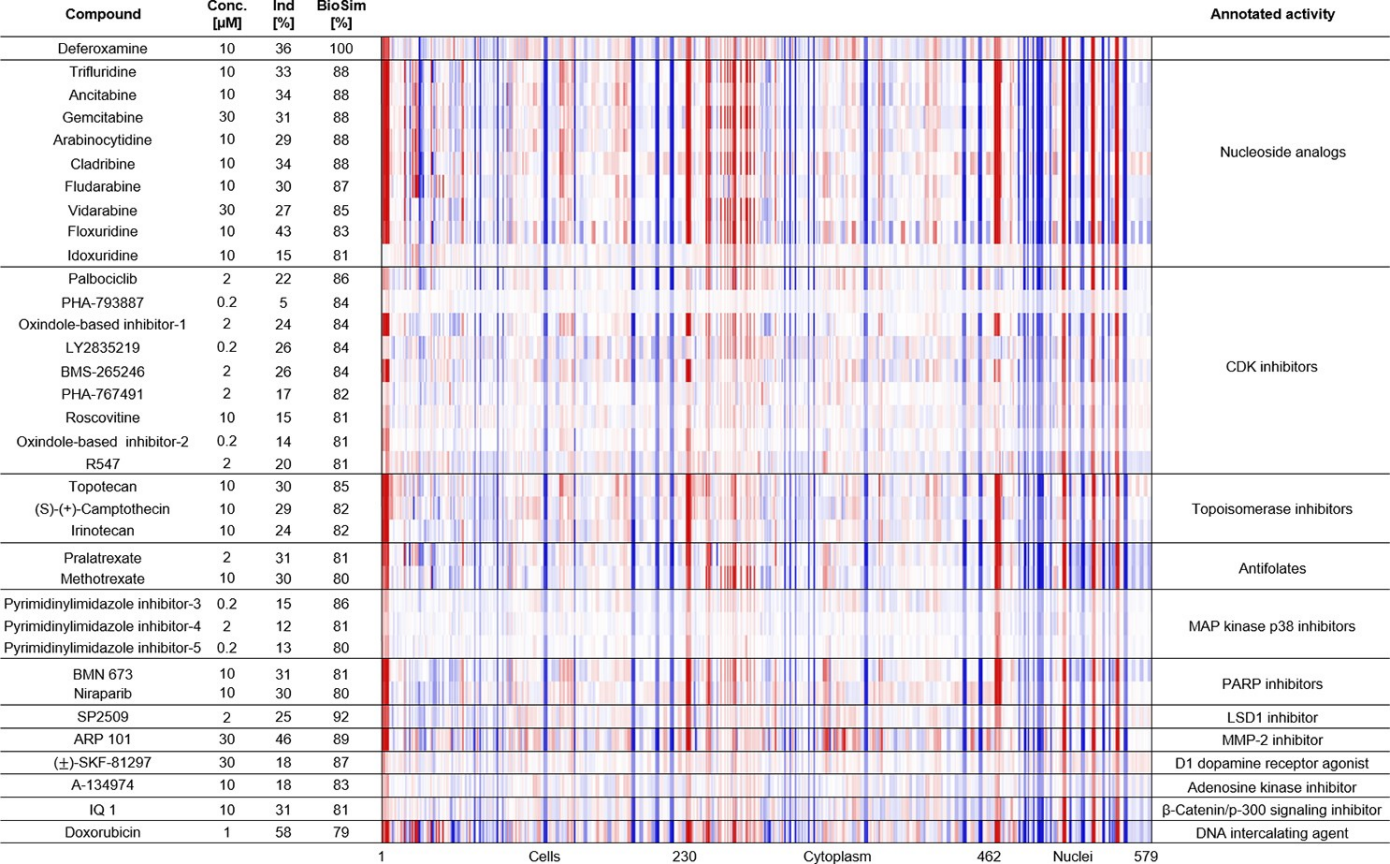
Morphological fingerprints of annotated references with high biosimilarity to DFO (>75 %). The top line of the heatmap profile is set as a reference fingerprint (100 % BioSim) to which the following fingerprints are compared. The set of 579 parameters is divided in parameters related to the cell (1–229), cytoplasm (230–461) and nuclei (462–579). Values were normalized to the DMSO control. Blue color: decreased parameter, red color: increased parameter. The structures of references not depicted in the main figures are shown in Table S4.

The fingerprints of more than 20 additional references displayed high similarity to DFO (BioSim between 79 and 92 %, Figure 3) and their annotated activity or targets were diverse‐nucleoside analogues, cyclin‐dependent kinases (CDKs), folic acid analogues, topoisomerase, poly(ADP‐Ribose)‐polymerase (PARP), lysine‐specific histone demethylase 1 (LSD1), matrix metalloproteinase‐2 (MMP‐2), dopamine 1 receptor, adenosine kinase, β‐catenin signaling or DNA intercalation. At first glance, these compounds do not share any common activity with iron chelators. However, iron is a crucial element involved in a wide range of cellular processes that are indispensable to life. Many enzymes involved in DNA synthesis and repair require iron as a cofactor^[10]^ and iron chelators are known to inhibit cell proliferation and induce cell‐cycle arrest in G1/S‐phase.^[11]^ Furthermore, iron chelators can influence the expression of several cyclins and cyclin‐dependent kinases, thereby leading to cell‐cycle arrest.^[23]^ Thus, the biosimilarity of iron‐chelating agents and nucleoside or folic acid analogues, topoisomerase‐, MAP kinase p38‐, CDK‐, PARP‐, LSD1‐ and MMP‐2 inhibitors and the D1 dopamine receptor agonist in the CPA is most likely not based on modulation of a similar target, that is, by direct interaction with proteins, but rather on a same MoA, that is, induction of cell‐cycle arrest.[^23^, ^24^]

**Figure 4:**
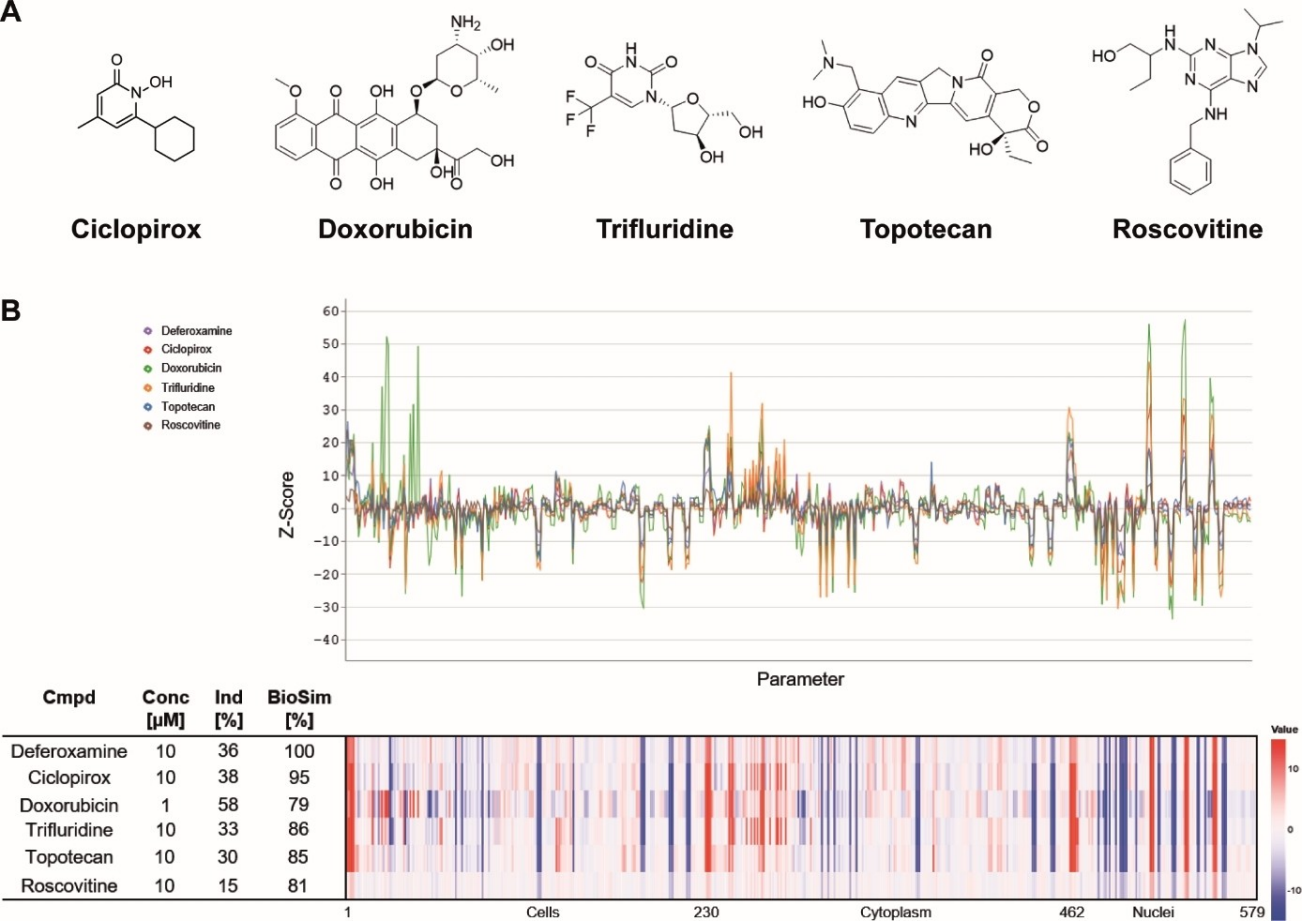
Selected references with a high biosimilarity to DFO. A) Structures and B) morphological fingerprint*s* of annotated reference compounds with high fingerprint similarity (BioSim >75 %) to 10 μM deferoxamine. The top line of the heatmap profile is set as a reference fingerprint (100 % BioSim) to which the following fingerprints are compared. The set of 579 parameters is divided in parameters related to the cell (1–229), cytoplasm (230–461) and nuclei (462–579). Values were normalized to the DMSO control. Blue: decreased parameter, red: increased parameter.

A representative selection of references with a high biosimilarity to DFO is depicted in Figure 4, including the kinase inhibitor roscovitine,^[25]^ the nucleoside analogue trifluridine,^[26]^ the DNA topoisomerase 1 inhibitor topotecan, the DNA intercalating agent doxorubicin and the iron chelator ciclopirox. Beyond the high biosimilarity to DFO (Figure 3), these references also exhibit a high compound cross‐similarity (Table S5) and define a CPA cluster based on a similar morphological phenotype.

**Figure 5:**
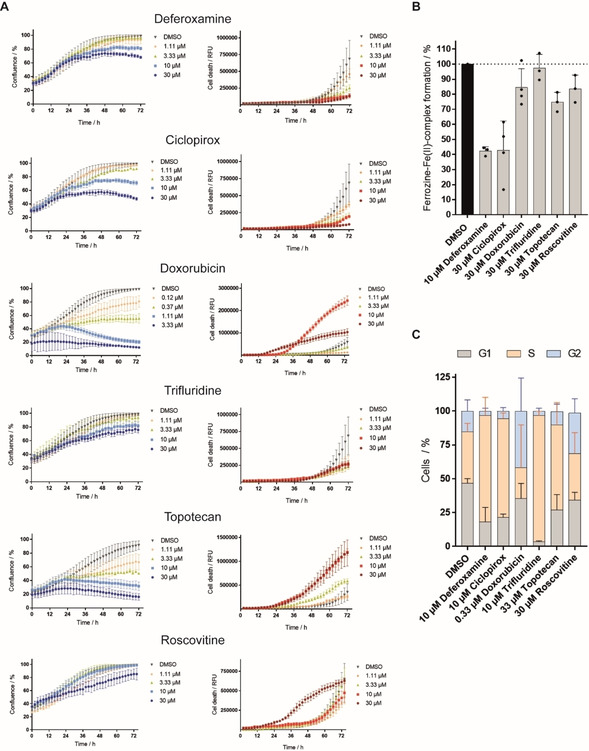
Influence of reference compounds with high fingerprint similarity to DFO on cell growth, iron chelation and the cell cycle. A) Influence of reference compounds on the growth behavior of U‐2OS cells. Cells were incubated with the compounds or DMSO as a control for 72 h and propidium iodide (PI) to detect dead cells. Images were acquired every 2 h by using the IncuCyte S3 imaging system. Image‐based analysis was used to quantify cell growth by means of cell confluence as readout, or dead cells by means of PI fluorescence. B) Iron chelation by reference compounds at 30 μM as determined by means of Ferrozine‐Fe^II^ complex formation. Data shown are mean±SD of three independent experiments. C) Influence of reference compounds on the cell cycle. U‐2OS cells were treated with compounds or DMSO as a control for 22 h and afterwards pulsed for another 2 h with 10 μM EdU (5‐ethynyl‐2’‐deoxyuridine) prior to fixation and staining of DNA with PI. Number of cells in S‐phase was determined by means of flow cytometry. Data shown are mean±SD of three independent experiments.

To confirm the underlying MoA of this cluster, the bioactivity of the selected references was further evaluated by means of real‐time live‐cell imaging in U‐2OS cells. All compounds impaired cell growth in a dose‐dependent manner (Figure 5A). Roscovitine was an exception as it only slightly decreased cell growth at 30 μM, while causing cell death, which was assessed by a propidium iodide (PI) stain. PI staining revealed toxic effects for doxorubicin‐treated cells at concentrations ≥10 μM and for topotecan‐treated cells at concentrations ≥3.33 μM. However, growth reduction under compound treatment with DFO, ciclopirox and trifluridine was not linked to cytotoxic effects delivering a first indication for cell‐cycle arrest. In addition, time‐resolved live‐cell imaging showed attenuated cell division as only a small number of cells underwent mitosis compared to the DMSO control (see Movies S1–S6). We then determined the iron‐chelating properties of the selected reference compounds at 30 μM and compared them to 10 μM DFO. Whereas DFO and ciclopirox restricted the formation of the Ferrozine‐Fe^II^ complex, which is indicative of iron (Fe^II^) chelation, trifluridine, roscovitine and topotecan did not influence complex formation (Figure 5B). To analyze the influence on the cell cycle, U‐2OS cells were treated for 22 h with the reference compounds and afterwards pulsed for another 2 h with the thymidine analogue 5‐ethynyl‐2’‐deoxyuridine (EdU), which is incorporated in the DNA during S phase. Detection of cells stained with PI and EdU demonstrated that DFO increased the number of cells in the S phase (Figure 5C, Table S6). In addition, ciclopirox, trifluridine and topotecan led to accumulation of cells in the S phase. Doxorubicin and roscovitine increased the number of cells with 4 N DNA content that may result from a G2 or M‐phase arrest or cytokinesis failure (Figure 5C, Table S6). Live‐cell imaging (Figure 5A) of doxorubicin‐ and roscovitine‐treated U‐2OS cells did not reveal any accumulation of round cells (indicative of mitotic cells and M‐phase arrest) or failed cytokinesis during the 24 h treatment and thus suggests arrest in G2 phase (Movies S1–S3).

Collectively, these findings illustrate that the high cross‐similarity within this cluster arises from the shared phenotype of cell‐cycle arrest in S or G2 phase although the references have different targets. Moreover, not characterized compounds with high biosimilarity to this cluster should most likely have a related MoA. Cytological and proteome profiling approaches[^8a^, ^27^] have been used to identify novel iron chelators and have provided first evidence for their clustering with DNA damaging agents.^[8a]^ Several annotated targets of the reference compounds investigated here are enzymes that require metal ion binding for their activity (Table S7). However, only LSD1 is dependent on Fe^II^,^[28]^ thus iron chelation alone cannot explain the biosimilarity within the cluster. We demonstrate that reference compounds that are biosimilar to DFO in the CPA like nucleoside and folic acid analogues, inhibitors of cyclin‐dependent kinases (CDKs), topoisomerase, poly(ADP‐ribose)‐polymerase (PARP), lysine‐specific histone demethylase 1 (LSD1) or DNA intercalators have the interference with DNA synthesis or cell cycle as a common denominator.[^24a^, ^24b^, ^24c^, ^24d^, ^24e^, ^24k^, ^29^] However, there is no direct link between MMP‐2, MAP kinase p38 and adenosine kinase inhibition or dopamine 1 receptor activation and cell‐cycle regulation. The activity of these references (i. e., ARP 101, pyrimidinylimidazole inhibitor‐3‐5, A‐134974 and (±)‐SKF‐81297) on the cell cycle might not be related to the nominal target, i. e., the target most commonly associated with the compound.^[30]^ The adenosine kinase inhibitor A‐134974 structurally belongs to the group of nucleoside analogues (Table S4). The dopamine 1 receptor agonist (±)‐SKF‐81297 also modulates the histone‐lysine‐N‐methyltransferase EHMT2 (also known as G9a) and lysine‐specific demethylase 4 A. MMP‐2 is hardly expressed in U‐2OS cells.^[31]^ Therefore, the activity in the CPA may be due to a different target. Moreover, the reference compounds inhibiting MAP kinase p38 and the adenosine kinase do not display a high cross‐similarity among their target class in the CPA also suggesting an activity due to a different target.

### Identification of uncharacterized compounds with high biosimilarity to the Fe‐chelation cluster

**Figure 6:**
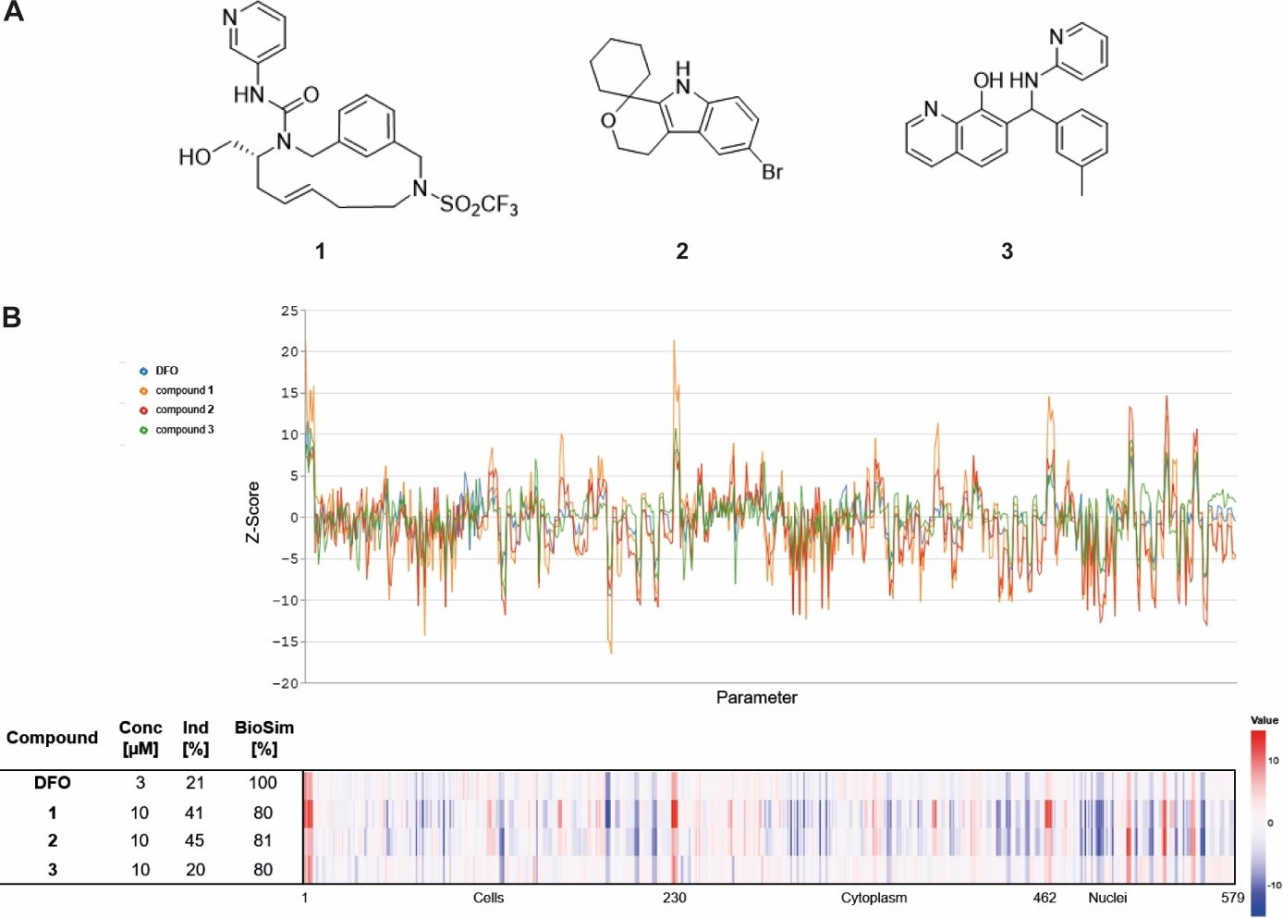
Research compounds with high biosimilarity to DFO. A) Structures of research compounds 1–3. B) Morphological fingerprints of research compounds 1–3 with high biosimilarity (BioSim >75 %) to 3 μM deferoxamine. The top line of the heatmap profile is set as a reference fingerprint (100 % BioSim) to which the following fingerprints are compared. The set of 579 parameters is divided in parameters related to the cell (1–229), cytoplasm (230–461) and nuclei (462–579). Values were normalized to the DMSO control. Blue: decreased parameter, red: increased parameter.

The confirmed MoA of the cluster allows the MoA prediction for uncharacterized compounds based on morphological fingerprint comparison. Therefore, we explored 9619 novel and structurally diverse natural product‐inspired compounds^[32]^ or pseudo‐natural products,^[33]^ which were synthesized in house, without annotated activity that showed biosimilarity to DFO. The natural product‐inspired compound **1** ^[34]^ and product **2** ^[35]^ exhibited a high biosimilarity (≥80 %) to 3 μM DFO (Figure 6A and B). In addition, several derivatives of 8‐hydroxyquinoline (compounds 3–9), which is a known metal‐chelating scaffold,[^10b^, ^36^] displayed similar CPA fingerprints to DFO (3 μM; Table S8, BioSim 76–89 %). Compound **3** was selected as a representative example for all 8‐hydroxyquinoline derivatives.

**Figure 7:**
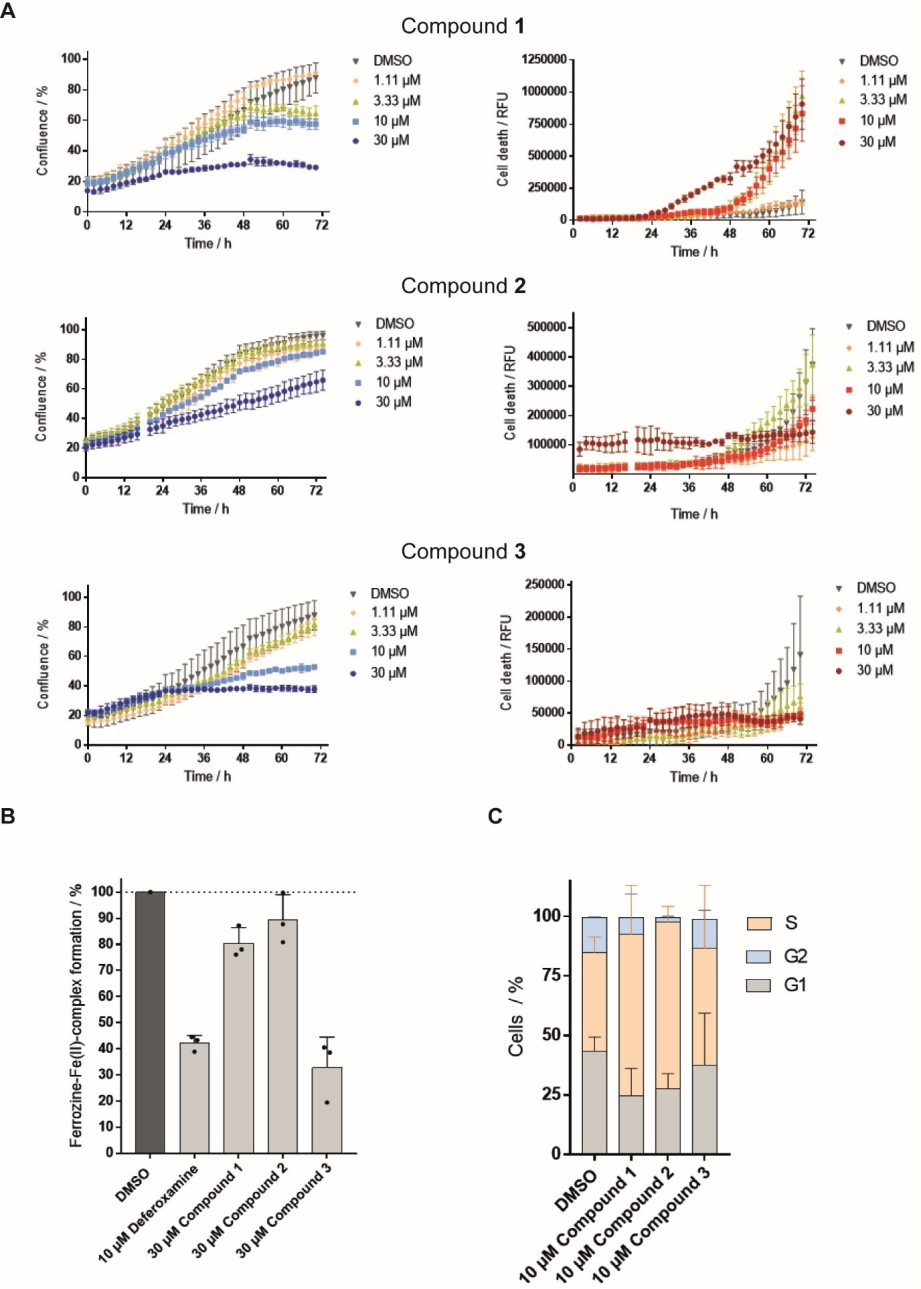
Influence of compounds **1**, **2** and **3** on cell growth, iron chelation and cell cycle. A) Influence of the compounds on the growth behavior of U‐2OS cells. Cells were incubated with the compounds or DMSO as a control for 72 h and propidium iodide (PI) to detect dead cells. Images were acquired every 2 h by using the IncuCyte S3 imaging system. Image‐based analysis was used to quantify cell growth by means of cell confluence as readout, or dead cells by means of PI fluorescence. B) Iron chelation by the compounds at 30 μM as determined by means of Ferrozine‐Fe^II^ complex formation. Data shown are mean values±SD of three independent experiments. C) Influence of the compounds on the cell cycle. U‐2OS cells were treated with the compounds or DMSO as a control for 22 h and afterwards pulsed for another 2 h with 10 μM EdU (5‐ethynyl‐2’‐deoxyuridine) prior to fixation and staining of DNA with PI. Number of cells in S‐phase was determined by means of flow cytometry. Data shown are mean values±SD of three independent experiments.

Exploration of the phenotype induced by compound **1**, **2** and the 8‐hydroxyquinoline derivative **3** revealed that all three compounds reduced the growth of U‐2OS cells in a concentration dependent manner (Figure 7A). Whereas no cell death was detectable for **2** and **3**, indicating cell‐cycle arrest, compound **1** was toxic after 24 h of treatment at concentrations≥3.33 μM (Figure 7A). 8‐hydroxyquinolines are known metal ion‐binding ligands with similar affinities for Fe^II^ and Fe^III^.^[20]^ Thus, compound **3**, as expected, chelates iron (Fe^II^ at 30 μM although the cheminformatics tools did not predict such activity (Table S9), while compound **1** and **2** did not affect Ferrozine‐Fe^II^ complex formation (Figure 7B). However, cell‐cycle analysis by means of EdU and PI staining revealed that all three compounds **1**–**3** at 10 μM indeed led to the accumulation of cells in the S‐phase (Figure 7C, Table S10).

To gain further insight into the possible mechanism of action, we explored the DNA‐binding activity of compounds **1** and **2**. However, **1** and **2** failed to displace the minor groove binder DAPI and the DNA intercalator propidium iodide and, thus, do not bind to DNA (Figure S1). In addition, compounds **1** and **2** were analyzed for modulation of topoisomerase 1 and 2 and selected CDK/cyclin complexes. 30 μM compound **1** and **2** neither inhibited the activity of both topoisomerases nor the activity of several CDK/cyclin complexes in biochemical assays (Tables S11–13). However, the compounds may inhibit these enzymes in cells or target different proteins that are involved in cell‐cycle regulation.

Macrocycle **1** ^[34]^ and the natural‐product‐inspired compound **2** do not chelate Fe^II^ but cause the accumulation of cells in S‐phase, which is in line with the observed activity in this cluster. Importantly, most compounds in this cluster did not impact cell growth after 22 h at a concentration, at which they increased the number of cells in S‐phase. Therefore, CPA enables the identification of small molecules that impair the cell cycle without marked decrease in cell growth at that stage and by employing staining for cellular components or compartments rather than using cell‐cycle‐specific markers.

We performed hierarchical clustering in order to investigate the ability of the cell painting assay to distinguish between different mechanism of action within the Fe/DNA synthesis cluster.

**Figure 8:**
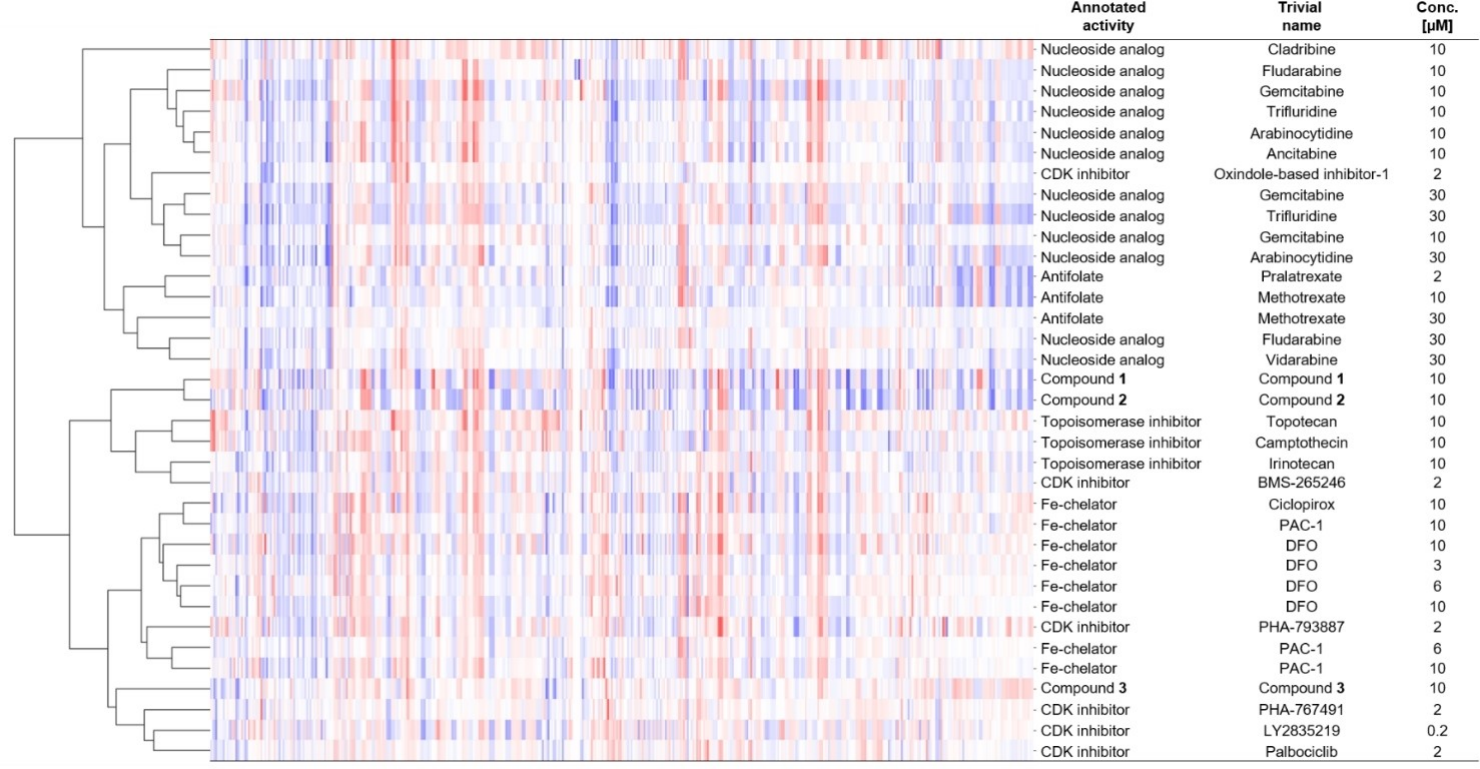
Hierarchical clustering of compounds with high biosimilarity (>80 %) to 10 μM DFO. Compounds with high fingerprint similarity (>80 %) including different compound batches and concentrations were subjected to hierarchical clustering. For this, the list of similar compounds was further filtered for compounds with an induction between 17 and 37 % to mitigate induction effects and was restricted to references with a reported mode of action that is shared by the Fe/DNA synthesis cluster. Structures are shown in Table S4.

The hierarchical clustering divided the cluster into two major groups (Figure 8). One group comprises the nucleoside analogues and antifolates, both functioning as mimetics of biological macromolecules. The second group covers the iron chelators, the topoisomerase inhibitors and the CDK inhibitors, which modulate protein activity. One exception is the oxindole‐based CDK inhibitor‐1 that represents an outlier as it is the only CDK inhibitor, which was assigned to the group of mimetics. Compounds **1**–**3** were assigned to the second subcluster of iron chelators, topoisomerase and CDK inhibitors. However, compound **3** is clearly separated from compounds **1** and **2** and clusters, as expected based on the Fe^II^ chelating activity (Figure 7B), together with the annotated iron chelating agents (Figure 8). This finding demonstrates that for compound **3** the cell painting assay would have been able to predict iron chelating activity completely independent of chemical similarity comparison. To generalize this prediction, more annotated iron chelators and cell‐cycle regulators would need to be screened and further evaluated. Noteworthy, the hierarchical clustering divided the cluster regarding the mechanism of action only when all six dyes were considered in the analysis. Hierarchical clustering solely based on DNA, actin and plasma membrane/Golgi staining did not lead to a meaningful separation into subclusters (Figure S2). This demonstrates the advantages of a multiplexed phenotypic profiling like the cell painting assay that not only enables the identification of compounds with a common mode of action but also offers insight into target‐related bioactivity, that is, mechanism of action.

## Conclusion

Iron chelators and compounds that impair the cell cycle in S/G2‐phase by targeting DNA or S/G2 regulating proteins display high biosimilarity in the cell painting assay and define a cluster that can be employed to predict a MoA for novel iron‐targeting agents and cell‐cycle modulators in general based on their morphological fingerprints. We identified three uncharacterized compounds that are biosimilar to this cluster and proved that they induce S‐phase arrest. In addition, we demonstrate that hierarchical clustering allows to distinguish between the different mechanisms of action. Our findings underscore the predictive value of unbiased morphological profiling for mode‐of‐action identification to shorten and render the MoA or target identification and validation process more efficient.

## Conflict of interest

The authors declare no conflict of interest.

## Supporting information

As a service to our authors and readers, this journal provides supporting information supplied by the authors. Such materials are peer reviewed and may be re‐organized for online delivery, but are not copy‐edited or typeset. Technical support issues arising from supporting information (other than missing files) should be addressed to the authors.

Supplementary

Supplementary

Supplementary

Supplementary

Supplementary

Supplementary

Supplementary
