# Supplementary material for: Morphological Profiling Identifies a Common Mode of Action for Small Molecules with Different Targets
Source: Chembiochem. 2020 Jul 24;21(22):3197–207. doi: 10.1002/cbic.202000381 (PMC7754162; doi:10.1002/cbic.202000381)
Supplement: Supplementary file 1 — Supplementary [file CBIC-21-3197-s001.pdf]

ChemBioChem

Supporting Information

Morphological Profiling Identifies a Common Mode of Action for Small Molecules with Different Targets

Tabea Schneidewind, Alexandra Brause, Axel Pahl, Annina Burhop, Tom Mejuch, Sonja Sievers, Herbert Waldmann,* and Slava Ziegler*

Supporting Information

Supplementary Figure S1-2, Movies S1-S3 and Tables S1-S13

Material and Experimental Section

Supplementary Figures

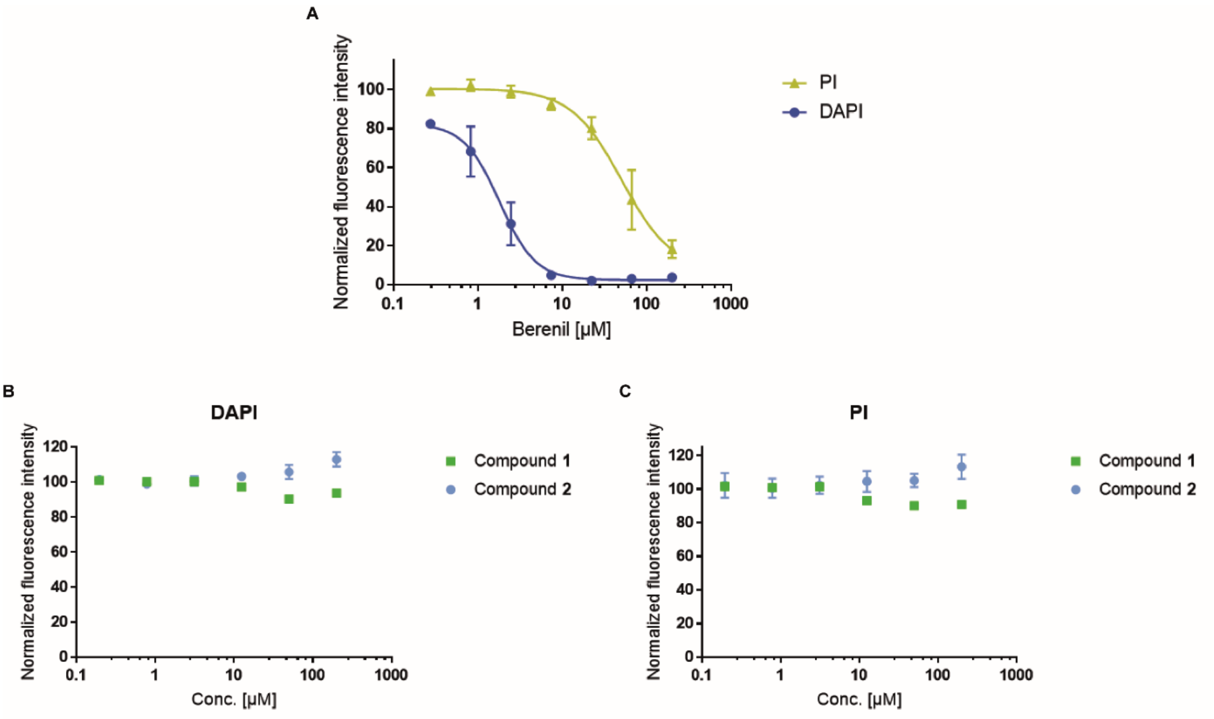

Figure S1: DNA binding of compound **1** and **2**. Calf thymus DNA was incubated with the minor groove binder DAPI and the intercalator PI in the presence of the positive control (A) Berenil or compound **1** and **2** (B+C). Binding of compound to the DNA would displace DAPI or PI depending on the binding mode and result in a decreased fluorescence signal.

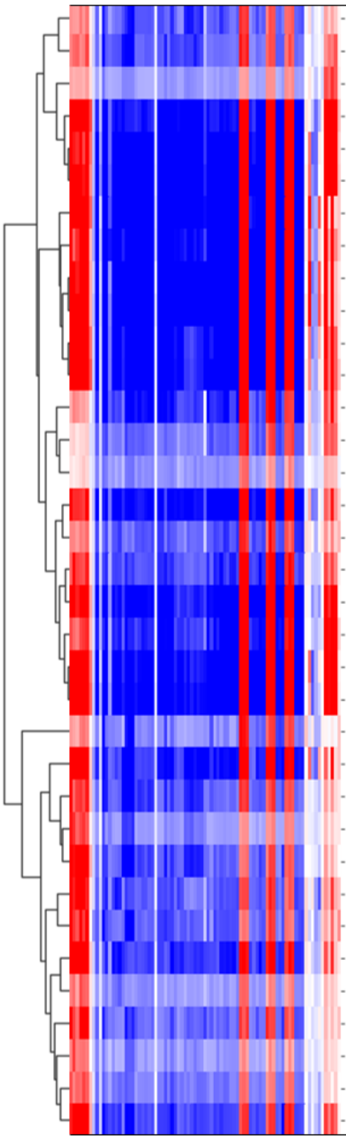

Annotated activity	Trivial name	Conc. [µM]
Fe-chelator	DFO	6
Fe-chelator	DFO	10
Antifolate	Methotrexate	30
Nucleoside analog	Vidarabine	30
Nucleoside analog	Fludarabine	30
Nucleoside analog	Gemcitabine	30
Nucleoside analog	Arabinocytidine	30
Nucleoside analog	Gemcitabine	10
Nucleoside analog	Fludarabine	10
Nucleoside analog	Gemcitabine	10
Nucleoside analog	Arabinocytidine	10
Nucleoside analog	Ancitabine	10
CDK inhibitor	Palbociclib	2
CDK inhibitor	LY2835219	0.2
CDK inhibitor	PHA-767491	2
Antifolate	Pralatrexate	2
Compound **2**	Compound **2**	10
Fe-chelator	DFO	10
Fe-chelator	Ciclopirox	10
Antifolate	Methotrexate	10
Nucleoside analog	Cladribine	10
Nucleoside analog	Trifluridine	10
CDK inhibitor	PHA-793887	2
Nucleoside analog	Trifluridine	30
Fe-chelator	PAC-1	10
Fe-chelator	PAC-1	6
Topoisomerase inhibitor	Irinotecan	10
Topoisomerase inhibitor	Camptothecin	10
Compound **1**	Compound **1**	10
Topoisomerase inhibitor	Topotecan	10
Compound **3**	Compound **3**	10
CDK inhibitor	BMS-265246	2
Fe-chelator	DFO	3
Fe-chelator	PAC-1	10
CDK inhibitor	Oxindole-based inhibitor-1	2

Figure S2: Hierarchical clustering of compounds with high biosimilarity (>80 %) to 10 µM DFO based on parameters that consider only the nucleus (Hoechst-33342) and cytoplasm (Phalloidin-Alexa Fluor 568 and WGA-Alexa Fluor 555) staining. Compounds with high fingerprint similarity (>80 %) including different compound batches and concentrations were subjected to hierarchical clustering. For this, the list of similar compounds was further filtered for compounds with an induction between 17 and 37 % to mitigate induction effects and was restricted to references with a reported mode of action that is shared by the Fe/DNA synthesis cluster.

Supplementary Movies

Supplementary Movie 1. U-2OS cells were treated with 0.3 % DMSO as a control

Supplementary Movie 2. U-2OS cells were treated with 0.37 µM Doxorubicin.

Supplementary Movie 3. U-2OS cells were treated with 30 µM Roscovitine.

Supplementary Movie 4. U-20S cells were treated with 10 µM Deferoxamine.

Supplementary Movie 5. U-2OS cells were treated with 30 µM Trifluridine.

Supplementary Movie 6. U-2OS cells were treated with 3.33 µM Topotecan.

Supplementary Tables

Table S1: Ion preferences of metal-chelating agents.

Chelating agent	Preference in binding metal ions
Deferoxamine	$Fe^{3+} >> Al^{3+} > Cu^{2+} > Zn^{2+} \approx Co^{2+} > Ni^{2+} \approx Fe^{2+} > Mg^{2+} > Ca^{2+}$ Sr^{2+}[1]
Ciclopirox	Fe^{3+}, trivalent > divalent[1f, 2]
1,10-Phenanthroline	Fe^{2+}[1e]<br>$Fe^{3+} >> Cu^{2+} \approx Ni^{2+} \approx Zn^{2+}$[3]
Catechol	Fe^{3+}, trivalent > divalent cations[1e, 4]

Table S2: Target prediction for DFO using web-based cheminformatic tools.[5]

Tool	Top predicted activity/target
PASS Online[6]	**Iron antagonist**<br>CDP-glycerol glycerophosphotransferase inhibitor<br>Polyamine-transporting ATPase inhibitor<br>Mucositis treatment<br>Antidote
Similarity ensemble approach (SEA)[7]	ATP-dependent molecular chaperone HSP82<br>Glutamate receptor 1<br>Deoxyhypusine synthase<br>Acetylpolyamine amidohydrolase<br>Putative agmatine deiminase
SwissTargetPrediction[8]	Matrix metalloproteinase 1<br>Endothelin-converting enzyme 1<br>Histone deacetylase 6<br>Histone deacetylase 2<br>Histone deacetylase 3/Nuclear receptor corepressor 2
SuperPred[9]	**Iron-chelating agent**<br>DNA topoisomerase II alpha<br>Hypoxia-inducible factor 1 alpha<br>DNA polymerase iota<br>Geminin
STITCH[10]	Transferrin receptor<br>Hypoxia-inducible factor 1 alpha<br>Solute carrier family 11 member<br>Aconitase 1<br>Tumor protein p53
PPB2[11]	Matrix metalloproteinase-2<br>Arachidonate 5-lipoxygenase<br>Cannabinoid CB1 receptor<br>Matrix metalloproteinase-3<br>Carbonic anhydrase II

Table S3: Target prediction for Ciclopirox and 1,10-phenanthroline using different web-based cheminformatic tools.[5]

Tool	Top predicted activity/target	
	Ciclopirox	1,10-Phenanthroline
PASS Online[6]	Antiseborrheic<br>Testosterone 17β-dehydrogenase inhibitor<br>Polarisation stimulant<br>CYP2J substrate<br>Membrane permeability inhibitor	Dehydro-L-gulonate decarboxylase inhibitor<br>Glutathione thiolesterase inhibitor<br>Amine dehydrogenase inhibitor<br>Taurine dehydrogenase inhibitor<br>Alkane 1-monooxygenase inhibitor
Similarity ensemble approach (SEA)[7]	Heat shock protein 90<br>Acetyl-CoA carboxylase<br>Isocitrate dehydrogenase<br>Taste receptor type 1	Heat shock protein 90<br>C-C chemokine receptor<br>Mitochondrial import inner membrane translocase<br>Neutrophil collagenase<br>Matrix metalloproteinase-9
SwissTarget Prediction[8]	Isocitrate dehydrogenase<br>Poly [ADP-ribose] polymerase 1<br>Dihydroorotate dehydrogenase<br>Cystic fibrosis transmembrane conductance regulator<br>Nitric oxide synthase	C-C chemokine receptor<br>Monoamine oxidase B<br>Indoleamine 2,3-dioxygenase<br>Quinone reductase 2<br>Serotonin 3a receptor
SuperPred[9]	Adenosine receptors<br>Adrenergic receptor<br>Angiotensin II type 2 receptor<br>Bradykinin B2 receptor<br>C-C chemokine receptor	Androgen receptor<br>Arachidonate 5-lipoxygenase<br>C-C chemokine receptor<br>Cytochrome P450<br>Estrogen receptor alpha
STITCH[10]	Prostaglandin-endoperoxide synthase<br>ATPase alpha 1 polypeptide<br>Arachidonate 15-lipoxygenase<br>Hypoxia inducible factor 1<br>Nuclear receptor subfamily 3	
PPB2[11]	Isocitrate dehydrogenase<br>11-beta-hydroxysteroid dehydrogenase<br>Phosphodiesterase 7A<br>Dopamine receptor<br>Adenosine receptor	Vascular endothelial growth factor receptor 2<br>Serotonin receptor<br>Nuclear factor NF-kappa-B p65 subunit<br>Kappa opiod receptor<br>Egl nine homolog 1

Table S4: Structures of reference compounds.

Trivial name	Structure	Trivial name	Structure
Ancitabine	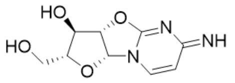	Palbociclib	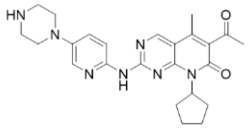
Gemcitabine	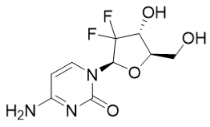	PHA-793887	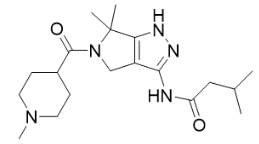
Arabinocytidine	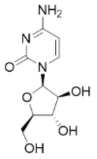	Oxindole-based inhibitor-1[12]	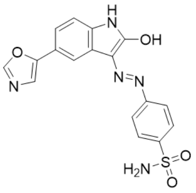
Cladribine	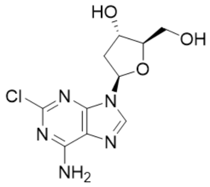	LY2835219	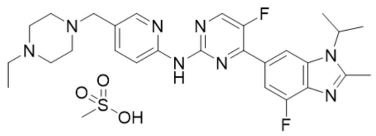
Fludarabine	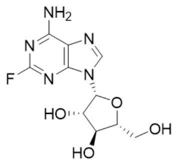	BMS-265246	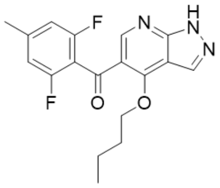
Vidarabine	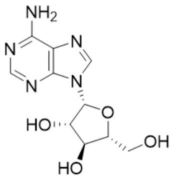	PHA-767491	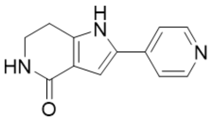
Floxuridine	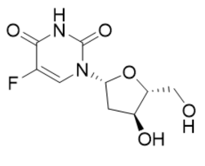	Oxindole-based inhibitor-2[12]	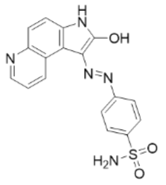
Idoxuridine	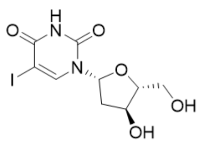	R547	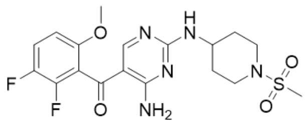

Trivial name	Structure	Trivial name	Structure
(S)-(+)-Camptothecin	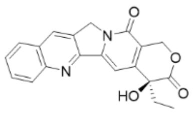	BMN-673	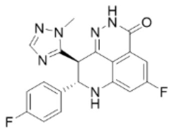
Irinotecan	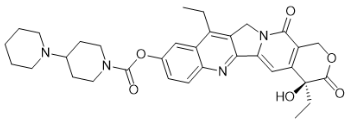	Niraparib	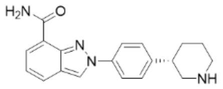
Pralatrexate	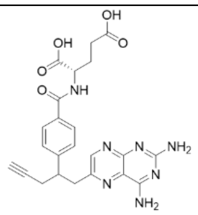	SP2509	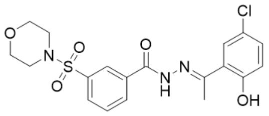
Methotrexate	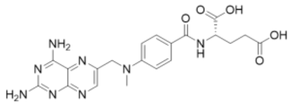	ARP 101	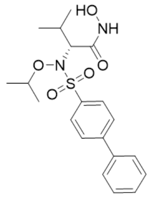
Pyrimidinylimidazole inhibitor-3[13]	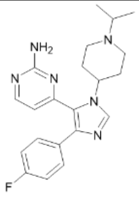	(±)-SKF-81297 hydrobromide	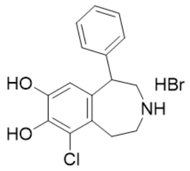
Pyrimidinylimidazole inhibitor-4[13]	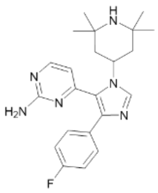	A-134974	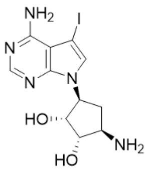
Pyrimidinylimidazole inhibitor-5[13]	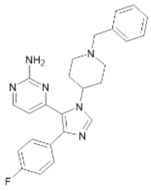	IQ-1	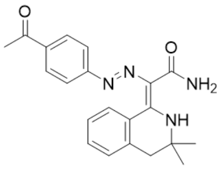

Table S5: Cross-similarity matrix between annotated references with morphological fingerprint similarity (>75 %) to 10 µM DFO. (see separate Excel file)

Table S6: Quantification of the percentage of U-2OS cells in the G1 (DNA content of N2), S and G2 (4N) phase of the cell cycle. Cells were treated with reference compounds or DMSO as a control for 22 h and afterwards pulsed for another 2 h with 10 µM EdU (5-ethynyl-2´-deoxyuridine) prior to fixation and staining of DNA with PI. DNA content and EdU incorporation was determined by means of flow cytometry; Data are mean of three independent experiments.

**Compound**	**Cells / %**					
	**G1**		**S**		**G2**	
	**Mean**	**SD**	**Mean**	**SD**	**Mean**	**SD**
DMSO	46.6	3.5	38.0	6.3	15.3	8.4
10 µM Deferoxamine	18.1	10.7	78.8	13.2	2.9	2.3
10 µM Ciclopirox	21.3	2.5	73.1	4.1	5.4	2.8
0.33 µM Doxorubicin	35.5	11.0	22.7	31.6	41.7	24.6
10 µM Trifluridine	3.5	0.7	93.2	3.2	3.1	2.4
3.33 µM Topotecan	26.8	11.4	62.9	16.5	9.9	5.5
30 µM Roscovitine	34.3	5.6	34.4	15.4	29.8	10.6

Table S7: Targets of biosimilar references to DFO that require metal ions for their activity.

**Target**	**Metal ion binding**
CDK, MAPK p38, AK	Mg^{2+}[14]
Topoisomerase	Mg^{2+}, Mn^{2+}, Ca^{2+}, Co^{2+}[15]
Poly (ADP-ribose) polymerase	Zn^{2+}[16]
Lysine specific demethylase 1	Fe^{2+}, Zn[17]
Metalloproteinase-2	Zn[18]

Table S8: 8-Hydroxyquinolines showing high biosimilarity (>76 %) to 3 µM DFO.

Compound	Concentration [µM]	Induction [%]	BioSim to 3 µM DFO [%]
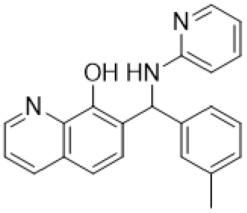 **3**	10	20	80
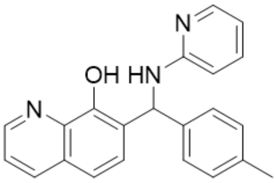 **4**	10	37	86
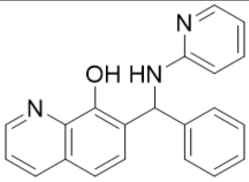 **5**	10	25	83
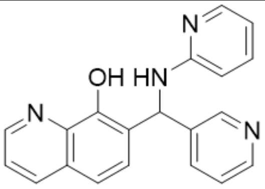 **6**	10	14	81
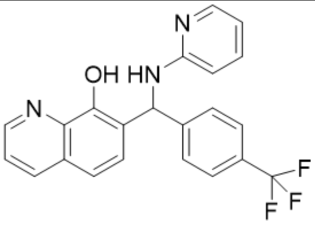 **7**	1	24	89

Compound	Concentration [µM]	Induction [%]	BioSim to 3 µM DFO [%]
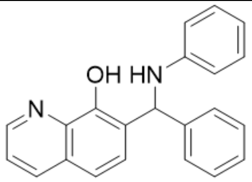 **8**	10	12	77
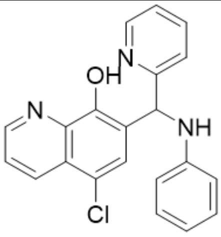 **9**	10	14	76

Table S9: Target prediction for 8-hydroxyquinoline using different web-based chemoinformatic tools.[5]

Tool	Top predicted activity/target
PASS Online	Corticosteroid side-chain-isomerase<br>Rhamnulose-1-phosphate aldolase<br>Glycosylphosphatidylinositol phospholipase D<br>Antiseborrheic<br>Magensium-protoprophyrin IX minimethyl ester cyclase
Similarity ensemble approach (SEA)	Bacterial leucyl aminopeptidase<br>Methionine aminopeptidase 2<br>Solute carrier family 40 member 1<br>NF-kappa-B inhibitor alpha<br>cAMP-specific 3’,5’-cyclic phosphodiesterase 4B
SwissTargetPrediction	Methionine aminopeptidase 2<br>Indoleamine 2,3-dioxygenase<br>Mannose-6-phosphate isomerase<br>Proteasome Macropain subunit MB1<br>Tryptophan 2,3-dioxygenase
SuperPred	Adenosine receptor<br>Adrenergetic receptor<br>Androgen receptor<br>Angiotensin II type 2 receptor<br>Arachidonate 5-lipoxygenase
STITCH	Glycerol dehydrogenase<br>Inner membrane protein<br>tRNA(Ile)-lysidine synthase<br>Quinone oxidoreductase
PPB2	Induced myeloid leukemia cell differentiation protein<br>Serotonin receptor<br>Kappa opioid receptor<br>Vascular endothelial growth factor receptor 2<br>P53-binding protein Mdm-2

Table S10: Quantification of the percentage of U-2OS cells in the G1 (DNA content of N2), S and G2 (4N) phase of the cell cycle. Cells were treated with compound **1**-**3** or DMSO as a control for 22 h and afterwards pulsed for another 2 h with 10 µM EdU (5-ethynyl-2´-deoxyuridine) prior to fixation and staining of DNA with PI. DNA content and EdU incorporation was determined by means of flow cytometry; Data are mean of three independent experiments.

**Compound**	**Cells / %**					
	**G1**		**S**		**G2**	
	**Mean**	**SD**	**Mean**	**SD**	**Mean**	**SD**
DMSO	43.2	6.1	41.5	6.6	14.8	0.2
10 µM Compound **1**	24.8	11.2	67.5	20.8	7.3	9.7
10 µM Compound **2**	27.6	6.3	69.8	6.7	2.2	0.3
10 µM Compound **3**	37.4	21.9	49.1	26.5	12.1	3.8

Table S11: Inhibition of human topoisomerase I relaxation by 30 µM compound **1** and **2**.

**Compound**	**% relaxed DNA Assay 1**	**% relaxed DNA Assay 2**	**% relaxed DNA Average**
Camptothecin, 10 µM	97.20	98.24	**97.72**
Camptothecin, 30 µM	94.06	94.42	**94.24**
Camptothecin, 50 µM	74.48	82.02	**78.25**
Camptothecin, 100 µM	37.15	45.70	**41.42**
Compound **1**, 30 µM	96.92	99.02	**97.97**
Compound **2**, 30 µM	98.48	99.30	**98.89**

Table S12: Inhibition of human topoisomerase IIα decatenation by 30 µM compound **1** and **2**.

**Compound**	**% decatenated DNA Assay 1**	**% decatenated DNA Assay 2**	**% decatenated DNA Average**
Etoposide, 10 µM	85.53	95.04	**90.28**
Etoposide, 30 µM	32.52	39.95	**36.23**
Etoposide, 50 µM	26.83	28.94	**27.89**
Etoposide, 100 µM	19.57	19.98	**19.77**
Compound **1**, 30 µM	102.40	97.00	**99.70**
Compound **2**, 30 µM	79.54	81.56	**80.55**

Table S13: Inhibition/Binding of selected CDK/cyclin complexes by 30 µM compound **1** and **2**.

Kinase	% Inhibition compound 1 (mean, n=2)	% Inhibition compound 2 (mean, n=2)	[ATP] / µM	Technology
CDK4/cyclin D1	8.5	-11	10	Adapta
CDK4/cyclin D3	-11	-12	10	Adapta
CDK6/cyclin D1	-13	-3	10	Adapta
CDK1/cyclin B	3.5	1	Km app	Z'Lyte
CDK2/cyclin A	2	0.5	Km app	Z'Lyte
CDK2/cyclin A1	5.5	4	-	LanthaScreen Binding
CDK2/cyclin E1	5	9	-	LanthaScreen Binding

Material and Experimental Section

Chemicals	Supplier	Product Number/CAS
DMEM medium (high glucose)	PAN Biotech	Cat# P04-03550
Fetal bovine serum	Gibco	Cat# 10500-084
Sodium pyruvate	PAN Biotech	Cat# P04-43100
Non-essential amino acids	PAN Biotech	Cat# P08-32100
Propidium iodide	Sigma Aldrich	Cat# P4864
DNase-free RNase A	Thermo Fisher Scientific	Cat# EN0531
Iron (II) sulfate heptahydrate	Sigma Aldrich	F8633
Ferrozine	Thermo Fisher Scientific	10522194
UltraPure™ Calf Thymus DNA Solution	Thermo Fisher Scientific	15633019
4’,6-Diamidin-2-phenylindole (DAPI)	Sigma Aldrich	D9542
Diminazene aceturate (Berenil)	Sigma Aldrich	D7770
**Commercial Kits**	**Supplier**	**Product Number**
EdU Click-it Plus AF488	Thermo Fisher Scientific	**C10632**
MycoAlert™ mycoplasma detection kit	Lonza	**LT07-318**
**Cell lines**	**Supplier**	
Human U-2OS cells (female)	CLS	Cat# 300364; RRID:CVCL_0042
**Software**		
FlowJo		RRID:SCR_008520<br>https://www.flowjo.com/solutions/flowjo
GraphPadPrism 6.0	GraphPad	RRID:SCR_002798<br>https://www.graphpad.com/scientific-software/prism/

Chemicals	Supplier	Product Number/CAS
IncuCyte Software	ESSEN BIOSCIENCE	https://www.essenbioscience.com/en/products/incucyte/
**Devices**		
LSRII flow cytometer	BD Biosciences	https://www.bdbiosciences.com/us/instruments/research/cell-analyzers/c/744788
IncuCyte S3	ESSEN BIOSCIENCE	https://www.essenbioscience.com/en/products/incucyte/
Sparks plate reader	Tecan	https://lifesciences.tecan.com/microplate-readers

Cell culture

The female human bone osteosarcoma cell line U-2OS were cultured in Dulbecco's Modified Eagle's medium (DMEM, high glucose) supplemented with 10 % fetal bovine serum, 4 mM L-glutamine, 1 mM sodium pyruvate and non-essential amino acids. The cells were maintained at 37 °C and 5 % CO_2 in humidified atmosphere. Mycoplasma tests were performed monthly using the MycoAltert™ Mycoplasma Detection Kit according to the manufacturer's instruction.

Cell painting assay

The described assay follows closely the method described by Bray et al.[19]

Initially, 5 µl U2OS medium were added to each well of a 384-well plate (PerkinElmer CellCarrier-384 Ultra). Subsequently, U2OS cell were seeded with a density of 1600 cells per well in 20 µl medium. The plate was incubated for 10 min at the ambient temperature, followed by an additional 4 h incubation (37 °C, 5 % CO2). Compound treatment was performed with the Echo 520 acoustic dispenser (Labcyte) at final concentrations of 10 µM, 3 µM or 1 µM. Incubation with compound was performed for 20 h (37 °C, 5 % CO2). Subsequently, mitochondria were stained with Mito Tracker Deep Red (Thermo Fisher Scientific, Cat. No. M22426). The Mito Tracker Deep Red stock solution (1 mM) was diluted to a final concentration of 100 nM in prewarmed medium. The medium was removed from the plate leaving 10 µl residual volume and 25 µl of the Mito Tracker solution were added to each well.

The plate was incubated for 30 min in darkness (37 °C, 5 % CO_2). To fix the cells 7 µl of 18.5 % formaldehyde in PBS were added, resulting in a final formaldehyde concentration of 3.7 %. Subsequently, the plate was incubated for another 20 min in darkness (RT) and washed three times with 70 µl of PBS. (Biotek Washer Elx405). Cells were permeabilized by addition of 25 µl 0.1 % Triton X-100 to each well, followed by 15 min incubation (RT) in darkness. The cells were washed three times with PBS leaving a final volume of 10 µl. To each well 25 µl of a staining solution were added, which contains 1 % BSA, 5 µl/mL Phalloidin (Thermo Fisher Scientific, A12381), 25 µg/ml Concanavalin A (Thermo Fisher Scientific, Cat. No. C11252), 5 µg/ml Hoechst 33342 (Sigma, Cat. No. B2261-25mg), 1.5 µg/ml WGA-Alexa594 conjugate (Thermo Fisher Scientific, Cat. No. W11262) and 1.5 µM µl/ml SYTO 14 solution (Thermo Fisher Scientific, Cat. No. S7576). The plate is incubated for 30 min (RT) in darkness and washed three times with 70 µl PBS. After the final washing step the PBS was not aspirated. The plates were sealed and centrifuged for 1 min at 500 rpm.

The plates were prepared in triplicates with shifted layouts to reduce plate effects and imaged using a Micro XL High-Content Screening System (Molecular Devices) in 5 channels (DAPI: Ex350-400/ Em410-480; FITC: Ex470-500/ Em510-540; Spectrum Gold: Ex520-545/ Em560-585; TxRed: Ex535-585/ Em600-650; Cy5: Ex605-650/ Em670-715) with 9 sites per well and 20x magnification (binning 2).

The generated images were processed with the CellProfiler package (https://cellprofiler.org/, version 3.0.0) on a computing cluster of the Max Planck Society to extract 1716 cell features (parameters) per microscope site. The data was then further aggregated as medians per well (9 sites -> 1 well), then over the three replicates.

Further analysis was performed with custom Python (https://www.python.org/) scripts using the Pandas (https://pandas.pydata.org/) and Dask (https://dask.org/) data processing libraries (separate publication to follow).

From the total set of 1716 parameters a subset of highly reproducible and robust parameters was determined using the procedure described by Woehrmann et al.[20] in the following way: Two biological repeats of one plate containing reference compounds were analysed. For every parameter, its full profile over each whole plate was calculated. If the profiles from the two repeats showed a similarity >= 0.8 (see below), the parameter was added to the set.

This procedure was only performed once and resulted in a set of 579 robust parameters out of the total of 1716 that was used for all further analyses.

To determine the phenotypic fingerprint for each test compound Z-scores were then calculated for each parameter as how many times the Median Absolute Deviation (MAD) of the controls the measured parameter value of a test compound deviates from the Median of the controls:

$$z - score = \frac{value_{meas.} - Median_{Controls}}{MAD_{Controls}}$$

The morphological compound fingerprint is then the list of z-scores of all parameters for one compound.

In addition to the morphological fingerprint, an induction value was determined for each compound as the fraction of significantly changed parameters, in percent:

$$Induction\ [\%] = \frac{number\ of\ parameters\ with\ abs.\ \ values > 3}{total\ number\ of\ parameters}$$

Similarities of morphological fingerprints were calculated from the correlation distances between two fingerprints (https://docs.scipy.org/doc/scipy/reference/generated/scipy.spatial.distance.correlation.html; Similarity = 1 - Correlation Distance) and the compounds with the most similar fingerprints

were determined from a set of 3000 reference compounds that was also measured in the assay.

Live-cell imaging

For live-cell imaging, 2,500 U-2OS cells were seeded per well in a 96-well plate and incubated overnight. Cells were then treated with different small molecules at the indicated concentrations or DMSO as a control in medium containing propidium iodide (16.67 µg/µL) to assess toxicity. Live-cell imaging was performed on the IncuCyte S3 microscope (Essen Bioscience, USA) using 10X objective with an image acquisition every two hours. Cell growth was assessed by means of confluence and toxicity by means of propidium iodide fluorescence as a readout through quantitative kinetic processing metrics obtained from the time-lapse image acquisition using the IncuCyte S3 software (2019).

Flow cytometry

1.25 x 10^5 U-2OS cells were seeded per well in 6–well plates and incubated overnight. The following day, cells were treated with the indicated compound concentrations or DMSO as a control for 22 hours. Afterwards cells were pulsed with 10 µM EdU (5-ethynyl-2´-deoxyuridine) or medium as a control for another two hours. Cells were washed with PBS, detached using trypsin and re-suspended in PBS. Cells were centrifuged at 1258 g for 5 min at room temperature and washed with 1 % BSA in PBS. Cells were fixed with 4 % PFA in PBS and subjected to a click-reaction to label the EdU with a fluorophore. Afterwards, cells were stained with a propidium iodide solution (100 µg/mL propidium iodide, 0.1 % (v/v) Triton X-100 and 100 µg/mL DNase-free RNase A in PBS) for 30 min at room temperature. Cell suspensions were filtered to FACS tubes through a nylon mesh before analysis. 10,000 cells for each sample were sorted by the BD LSRII analyzer (Becton Dickinson, USA). FlowJo 10.6.1 software was used for quantification and analysis of all data. For every analysis, FSC and SSC (forward and side scatter, respectively) gating was performed to exclude doublets and debris and to select single cells. All results were plotted using GraphPad Prism 6 software.

Iron chelation

12.5 µM Fe (II) was incubated with compounds at indicated concentrations or DMSO, deferoxamine or EDTA as controls for 10 min at room temperature. Afterwards, 0.5 mM Ferrozine was added and absorbance at 561 nm was detected with a plate reader using a clear 96-well plate.

DNA binding assay

The binding of small molecules to DNA was assessed by performing a competition assay using DAPI as a minor groove binder and PI as a competitor for the binding mode of DNA intercalation. Binding of a compound to DNA would displace DAPI or PI depending on the binding mode and result in a decreased fluorescence signal. Therefor, 1 µg calf thymus DNA was incubated with different concentrations of the compound and either 0.625 µM DAPI or 0.625 µM PI in DNA binding buffer (2 mM HEPES, 9.4 mM NaCl, 0.01 mM EDTA, pH 7.0). Fluorescence was measured immediately. The assay was performed in technical triplicates in black 96-well plates with clear bottom using 100 µL per well. First the fluorescence of DAPI (Ex/Em: 350/470 nm) and afterwards the fluorescence of PI (Ex/Em: 535/617 nm) was measured using a plate reader.

Topoisomerase assays performed by Inspiralis

In all experiments, the activity of the enzyme was determined prior to the testing of the compounds and 1 U defined as the amount of enzyme required to fully decatenate or relax the substrate. Compounds were tested at a fixed concentration of 30 µM and added to the reaction before the addition of the enzyme. Final DMSO concentration in the assays was 10 % (v/v).

Human Topoisomerase I relaxation assay

1 U of human topo I was incubated with 0.5 µg supercoiled plasmid DNA (pBR322) in a 30 µl reaction at 37°C for 30 min under the following conditions: 20 mM Tris HCl (pH 7.5), 200 mM NaCl, 0.25 mM EDTA and 5 % glycerol plus 10 % DMSO. Each reaction was stopped by the

addition of 30 µl chloroform/iso-amyl alcohol (24:1) and 30 µl Stop Dye (40 % sucrose, 100 mM Tris.HCl (pH 7.5), 10 mM EDTA, 0.5 µg/ml bromophenol blue), before being loaded on a 1.0 % TAE (Tris.acetate 0.04 mM, EDTA 0.002 mM) gel and run at 80V for 2 hours.

Topoisomerase II (alpha) decatenation assay

1 U of human topo IIα was incubated with 200 ng kinetoplast DNA in a 30 µl reaction at 37°C for 30 min under the following conditions: 50 mM Tris HCl (pH 7.5), 125 mM NaCl, 10 mM $MgCl_2$, 5 mM DTT, 0.5 mM EDTA, 0.1 mg/ml bovine serum albumin (BSA) and 1 mM ATP in 10 % DMSO. Each reaction was stopped by the addition of 30 µl chloroform/iso-amyl alcohol (24:1) and 30 µl Stop Dye (40 % sucrose, 100 mM Tris.HCl (pH 7.5), 10 mM EDTA, 0.5 µg/ml bromophenol blue), before being loaded on a 1.0 % TAE (Tris.acetate 0.04 mM, EDTA 0.002 mM) Gels run at 80V for 2 hours.

Data acquisition and analysis

Bands were visualised by ethidium staining for 10 min, destained for 10 min in water and analysed by gel documentation equipment (Syngene, Cambridge, UK) and quantitated using Syngene Gene Tools software. Raw gel data (fluorescent band volumes) collected from Syngene, GeneTools gel analysis software were calculated as a % of the 100 % control (the fully supercoiled or decatenated band) and converted to % inhibition. The raw gel data was analyzed using SigmaPlot Version 13 (2015). The global curve fit non-linear regression tool was used to calculate IC50 data using the following equation: Exponential Decay, Single, 2 Parameter $f = a*\exp(-b*x)$

Screening of CDK/cyclin complexes

The screening of selected CDK/cyclin complexes was performed by SelectScreen Kinase Profiling Service of Life Technologies according to the instructions provided on the company's website: https://www.thermofisher.com/de/de/home/products-and-services/services/custom-

services/screening-and-profiling-services/selectscreen-profiling-service/selectscreen-kinase-profiling-service.html

Hierarchical clustering

The hierarchical clustering was performed and visualized using the clustermap tool from the seaborn package.[21] The tool in turn uses the hierarchical clustering module from the scipy package.[22] The linkage method was "complete", the used metric was "correlation" which corresponds to the similarity measure used for profile comparison.
The clustermaps were either generated from the full parameter profiles or, to improve the visibility of less pronounced areas of the profiles, by forming sub-profiles in the following way: For all considered profiles, keep only those parameters from the profile, where all of the absolute values over all the rows are less than 10.0.

PseudoCode:

```
cutoff_filter = 10.0
    parameters_to_keep = []
    for parameter in ALL_PARAMETERS:
        absmax =
abs(dataframe_with_considered_profiles[parameter]).max()
        if absmax < cutoff_filter:
            parameters_to_keep.append(parameter)
```

Quantification and statistical analysis

Data from independent experiments (n) are presented as mean values ± standard deviation (SD). *N* is the number of technical replicates and *n* is the number of biological replicates. Data fitting was performed using GraphPad Prism 6.0. Details to descriptive quantifications can be found in figures, their respective legends and tables.

References

[1] a) G. Liu, P. Men, G. Perry, M. A. Smith, in *Free Radicals and Antioxidant Protocols* (Eds.: R. M. Uppu, S. N. Murthy, W. A. Pryor, N. L. Parinandi), Humana Press, Totowa, NJ, **2010**, pp. 123; b) H. Keberle, *Annals of the New York Academy of Sciences* **1964**, *119*, 758; c) K. H. Maclean, J. L. Cleveland, J. B. Porter, *Blood* **2001**, *98*, 3831; d) G. Crisponi, V. M. Nurchi, M. Crespo-Alonso, G. Sanna, M. A. Zoroddu, G. Alberti, R. Biesuz, *PLoS One* **2015**, *10*, e0133050; e) G. Crisponi, V. Nurchi, J. Lachowicz, *Metal ions in life sciences* **2019**, *19*; f) T. Linden, D. M. Katschinski, K. Eckhardt, A. Scheid, H. Pagel, R. H. Wenger, *The FASEB Journal* **2003**, *17*, 761.

[2] a) A. Subissi, D. Monti, G. Togni, F. Mailland, *Drugs* **2010**, *70*, 2133; b) M. Bohn, K. T. Kraemer, *Journal of the American Academy of Dermatology* **2000**, *43*, S57.

[3] M. McCann, A. Kellett, K. Kavanagh, M. Devereux, A. L. S. Santos, *Current Medicinal Chemistry* **2012**, *19*, 2703.

[4] R. Hider, *Thallasemia Reports* **2014**, *4:2261*.

[5] *Systems Chemical Biology - Methods and Protocols*, Humana Press, **2019**.

[6] a) http://www.pharmaexpert.ru/passonline/; b) A. Lagunin, A. Stepanchikova, D. Filimonov, V. Poroikov, *Bioinformatics* **2000**, *16*, 747.

[7] a) http://sea.bkslab.org/; b) M. J. Keiser, B. L. Roth, B. N. Armbruster, P. Ernsberger, J. J. Irwin, B. K. Shoichet, *Nature Biotechnology* **2007**, *25*, 197.

[8] a) http://www.swisstargetprediction.ch/; b) A. Daina, O. Michielin, V. Zoete, *Nucleic Acids Research* **2019**, *47*, W357.

[9] a) http://prediction.charite.de/index.php?site=home; b) J. Nickel, B.-O. Gohlke, J. Erehman, P. Banerjee, W. W. Rong, A. Goede, M. Dunkel, R. Preissner, *Nucleic Acids Research* **2014**, *42*, W26.

[10] a) http://stitch.embl.de/; b) M. Kuhn, C. von Mering, M. Campillos, L. J. Jensen, P. Bork, *Nucleic Acids Research* **2007**, *36*, D684.

[11] a) http://ppb2.gdb.tools/; b) M. Awale, J. L. Reymond, *Methods Mol Biol* **2019**, *1888*, 255.

[12] H. N. Bramson, J. Corona, S. T. Davis, S. H. Dickerson, M. Edelstein, S. V. Frye, R. T. Gampe, P. A. Harris, A. Hassell, W. D. Holmes, R. N. Hunter, K. E. Lackey, B. Lovejoy, M. J. Luzzio, V. Montana, W. J. Rocque, D. Rusnak, L. Shewchuk, J. M. Veal, D. H. Walker, L. F. Kuyper, *Journal of Medicinal Chemistry* **2001**, *44*, 4339.

[13] J. L. Adams, J. C. Boehm, T. F. Gallagher, S. Kassis, E. F. Webb, R. Hall, M. Sorenson, R. Garigipati, D. E. Griswold, J. C. Lee, *Bioorganic & Medicinal Chemistry Letters* **2001**, *11*, 2867.

[14] a) D. M. Jacobsen, Z.-Q. Bao, P. O'Brien, C. L. Brooks, M. A. Young, *Journal of the American Chemical Society* **2012**, *134*, 15357; b) L. Yu, L. Xu, M. Xu, B. Wan, L. Yu, Q. Huang, *Molecular Simulation* **2011**, *37*, 1143; c) Z. Wang, P. A. Cole, *Methods Enzymol* **2014**, *548*, 1.
[15] a) J. E. Deweese, N. Osheroff, *Metallomics* **2010**, *2*, 450; b) C. Sissi, M. Palumbo, *Nucleic acids research* **2009**, *37*, 702.
[16] a) C. Hegedűs, L. Virág, *Redox Biology* **2014**, *2*, 978; b) G. Gradwohl, J. M. Ménissier de Murcia, M. Molinete, F. Simonin, M. Koken, J. H. Hoeijmakers, G. de Murcia, *P Natl Acad Sci USA* **1990**, *87*, 2990.
[17] R. Anand, R. Marmorstein, *Journal of Biological Chemistry* **2007**, *282*, 35425.
[18] C. Yao, B. Chen, Z. Kang, Y. Liu, X. Qi, Q. Wang, *Journal of Biomolecular Structure and Dynamics* **2020**, *38*, 275.
[19] M. A. Bray, S. Singh, H. Han, C. T. Davis, B. Borgeson, C. Hartland, M. Kost-Alimova, S. M. Gustafsdottir, C. C. Gibson, A. E. Carpenter, *Nat Protoc* **2016**, *11*, 1757.
[20] M. H. Woehrmann, W. M. Bray, J. K. Durbin, S. C. Nisam, A. K. Michael, E. Glassey, J. M. Stuart, R. S. Lokey, *Molecular BioSystems* **2013**, *9*, 2604.
[21] https://seaborn.pydata.org/generated/seaborn.clustermap.html.
[22] https://docs.scipy.org/doc/scipy/reference/cluster.hierarchy.html#module-scipy.cluster.hierarchy.
